# The pretender of Parkinson's disease: What neurologists need to know about functional movement disorders

**DOI:** 10.1111/cns.13705

**Published:** 2021-07-13

**Authors:** Xiaodi Hao, Jiewen Zhang, Yue Huang

**Affiliations:** ^1^ Department of Neurology Zhengzhou University People's Hospital Zhengzhou China

**Keywords:** dissociative conversion disorder, functional movement disorders, parkinsonism, psychological parkinsonism

## CONFLICTS OF INTEREST

The authors declare that the research was conducted in the absence of any commercial or financial relationships that could be construed as a potential conflict of interest. The authors declare no conflict of interest.

Dear editors,

Parkinsonism is a clinical syndrome characterized by tremor, rigidity, akinesia, and postural disturbances. Parkinson's disease is the most common cause of parkinsonism, but there are numerous other causes. Functional movement disorders (FMDs) comprise a broad range of clinical symptoms not explained by a classical neurological disease. Tremor is the most common symptom in FMDs^1^, ^2^ followed by dystonia, myoclonus, balance disorder, bradykinesia, cranial movement, hemifacial spasms, restless legs, and so on. FMDs are often categorized as “psychogenic,” and the psychological parkinsonism is about 10% of patients with FMDs.^3^

A 53‐year‐old woman presented with a 2‐year history of bradykinesia and rigidity. This patient presented with cogwheel rigidity, shortened stride length, and a decrease in arms swing. But she had a good balance with no difficulty in turns and no decreased facial expressions. She claimed to have an operation because of low blood pressure 2 years ago. Orthostatic hypotension was not found on our examinations and external anal sphincter‐electromyography was normal to exclude multiple system atrophy. The results of routine laboratory tests were normal, as well as the brain MRI. Also, her cerebrospinal fluid (CSF) revealed a normal leukocyte count, protein level, immunoglobulin G index, and no unique oligoclonal bands. Her condition got deteriorated after dopa treatment. Interestingly, her bradykinesia could be resolved after a specific action by herself, and her symptoms almost completely reversed after only 1 week of placebo. This patient was diagnosed with dissociative conversion disorder by a professional psychologist finally.

It is important to differentiate FMDs from Parkinson's disease or other neurodegenerative diseases, as the latter often progresses inexorably over a period of years. Although ignoring a suspicion of psychological disorder seems more acceptable than a diagnosis of organic disease, there are many clues to the FMDs, like the history, physical examination, laboratory assessment, and treatment response. Distractibility, abrupt onset, variability of manifestation over time, and selective disabilities are common clinical characteristics in FMDs.^1^ Diagnosing patients with psychological movement disorders often met with anger. While functional, sometimes interchangeably with “psychological,” is the term used in the diagnostic and statistical manual of mental disorders (DSM‐5) and seems more acceptable to patients. The DSM‐5 added a clinical criterion in 2013, which allows a ‘‘rule‐in’’ procedure in firming a diagnosis of FMDs so that it is no longer considered a diagnosis of exclusion.^2^

Functional movement disorders account for even 15% of neurology outpatient clinics, which are more common in women^1^, ^2^ and range from 17 to 83 years old.^1^ The incidence of functional neurological disorders is 4 to 12 of 100,000 per year.^2^ Definitely, the etiology of FMDs is multifactorial. The bio‐psycho‐social model is considered more convincing to understand the underlying mechanisms in FMDs. Although no specific gene has been associated with FMDs, there is a possible role of certain forms of genetic variants in predisposition to different movement disorders, like Potassium (K+) Channel Tetramerization Domain (KCTD)‐related family and catechol O‐methyltransferase (COMT) variants.^4^, ^5^ Increasing neuroimaging studies identified the hypoactivation of the cortical and subcortical motor pathways and increased modulation by the limbic system in FMDs, like strengthened connectivity between the limbic, cognitive, and motor networks in patient with FMDs.^5^ These abnormal cerebral regions were thought related to motor planning and execution.^6^ With F‐dopa, PET is helpful in distinguishing FMDs from Parkinson's disease in which abnormalities of fluorine accumulation are typically pronounced in the putamen and spared in the caudate.^3^, ^7^ A growing body of evidence suggests that physical trauma or stressful event often precedes or triggers FMDs, like motor vehicle accidents, operations, or fractures.^8^ Their pathogenic mechanisms may be related to the overactivity of brain networks reported in patients with functional neurological disorders, including energy regulation, threat detection, and action preparation. Certainly, some patients did present with combined FMDs and organic movement disorders, which is about 10%.^9^

Functional movement disorders still remain an enormous therapeutic challenge. Patients present for repeated medical attention because their physical suffering is real. Currently, there is no consensus about the treatment of FMDs, psychotherapy and psychoactive medications are typically early treatment attempts. Although a striking improvement can be observed in FMDs patients with successful treatment, movement disorders often have poor outcomes in those persisting beyond 6 months.^1^ The purpose of this study was to provide more data on functional movement disorder to emphasize its importance and to help reach a correct diagnosis.

## Data Availability

The datasets are available from the corresponding author on reasonable request.
